# Mechano-logical model of *C. elegans* germ line suggests feedback on the cell cycle

**DOI:** 10.1242/dev.126359

**Published:** 2015-11-15

**Authors:** Kathryn Atwell, Zhao Qin, David Gavaghan, Hillel Kugler, E. Jane Albert Hubbard, James M. Osborne

**Affiliations:** 1 Computational Biology Group, Department of Computer Science, University of Oxford, Oxford OX1 3QD, UK; 2 Biological Computation Group, Computational Science Laboratory, Microsoft Research Cambridge, Cambridge CB1 2FB, UK; 3 Skirball Institute of Biomolecular Medicine, Department of Cell Biology andKimmel Center for Stem Cell Biology, New York University School of Medicine, New York, NY 10016, USA; 4 Faculty of Engineering, Bar-Ilan University, Ramat Gan 5290002, Israel; 5 School of Mathematics and Statistics, University of Melbourne, Melbourne 3010, Australia

**Keywords:** *C. elegans*, Contact inhibition, Germ line, Mechanics, Modeling, Stem cell

## Abstract

The *Caenorhabditis* *elegans* germ line is an outstanding model system in which to study the control of cell division and differentiation. Although many of the molecules that regulate germ cell proliferation and fate decisions have been identified, how these signals interact with cellular dynamics and physical forces within the gonad remains poorly understood. We therefore developed a dynamic, 3D *in silico* model of the *C. elegans* germ line, incorporating both the mechanical interactions between cells and the decision-making processes within cells. Our model successfully reproduces key features of the germ line during development and adulthood, including a reasonable ovulation rate, correct sperm count, and appropriate organization of the germ line into stably maintained zones. The model highlights a previously overlooked way in which germ cell pressure may influence gonadogenesis, and also predicts that adult germ cells might be subject to mechanical feedback on the cell cycle akin to contact inhibition. We provide experimental data consistent with the latter hypothesis. Finally, we present cell trajectories and ancestry recorded over the course of a simulation. The novel approaches and software described here link mechanics and cellular decision-making, and are applicable to modeling other developmental and stem cell systems.

## Abstract (precis)

**Summary:** A three dimensional *in silico* model of *C. elegans* predicts that adult germ cells are subject to a mechanical feedback on the cell cycle akin to contact inhibition.

## INTRODUCTION

Controlled cell proliferation and fate decisions underlie development, tissue maintenance, regeneration and repair. Although tremendous progress has been made identifying the molecular pathways that regulate division and differentiation in individual cells, less is known about how the behavior of cell populations is coordinated within a developing organ. During organogenesis, cells are influenced by a complex interplay between intrinsic molecular processes, external signals and mechanical forces. Unraveling the contribution of each component is experimentally challenging.

**Fig. 1.**
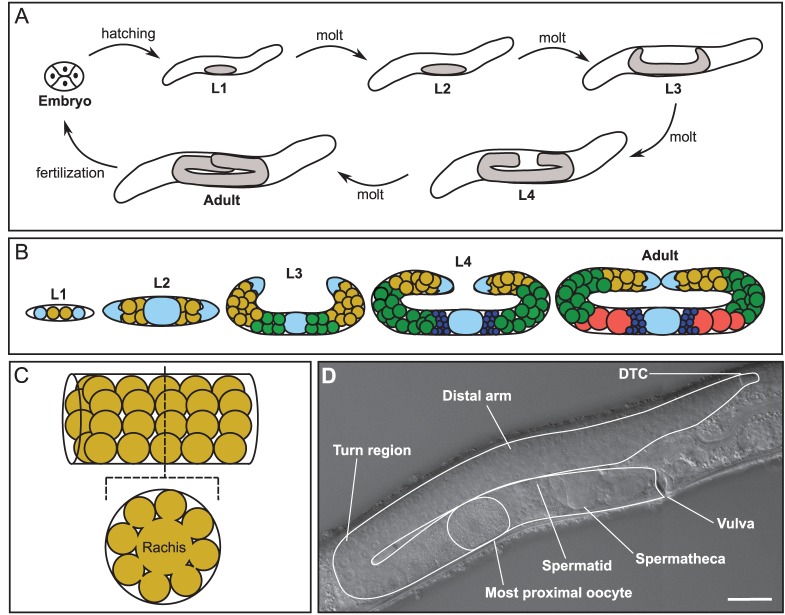
***C. elegans* germline development and organization.** (A) The *C. elegans* life cycle. Larval development is subdivided into four stages; at each stage the growing gonad is indicated in gray (not to scale). (B) A cartoon of germline development within the gonad (not to scale, under-represented cell counts from L2 onwards). The DTCs and somatic gonadal tissues are pale blue, with the central oval representing multiple cells and sheath cells omitted. Germ cells are color coded as follows: proliferating and meiotic S cells are yellow, meiotic cells are green, sperm are dark blue, and oocytes are red. (C) A cartoon depicting germ cell connections to the rachis. (D) Micrograph (composite of a distal and a proximal image) of a single early adult gonad arm, for comparison with drawings. The gonad arm and proximal-most oocyte are outlined. When the first oocyte is ovulated, sperm are pushed into the spermatheca. Scale bar: 25 µm.

Here, we present a computational model of *C. elegans* germline development and maintenance, a practical experimental system. Hermaphrodite gonadogenesis is summarized in Fig. 1, and takes place primarily over the larval life cycle stages L1-L4 (Fig. 1A). Our simulations begin immediately after the establishment of two separate gonad arms at the end of L2 (Fig. 1B). A distal tip cell (DTC), positioned at the end of each gonad arm, performs leader cell and signaling roles, both during gonadogenesis and in adulthood (Kimble and Hirsh, 1979; Kimble and White, 1981).

During the L3 and L4 larval stages, germ cells rapidly divide. The pressure generated by these divisions contributes to the anterior-posterior growth of the organ, as does active DTC migration (Kimble and White, 1981; Killian and Hubbard, 2005). As the DTCs move further from the center of the animal, proximal germ cells go out of range of their proliferation-promoting/differentiation-inhibiting signal and enter meiosis (Fig. 1B, green cells). During L4, the proximal-most meiotic cells differentiate as spermatocytes, each producing four sperm. In adults, oogenic germ cells either undergo apoptosis in the turn or develop into oocytes (Gumienny et al., 1999). With the exception of spermatogonia, sperm and the proximal-most oocytes, ‘germ cells’ are technically syncytial, as they retain a small opening onto the rachis, a central cytoplasmic reservoir that streams material into maturing oocytes (Fig. 1C) (Wolke et al., 2007). However, because germ nuclei are surrounded by their own cytoplasm and do not appear to share cytoplasmic components, they are referred to as ‘germ cells’ (Hirsh et al., 1976).

Germ cells are prevented from entering prophase of meiosis I within the first ∼13 cell diameters (CD) of the DTC in L3 larvae (20-25CD in adults) (Hansen et al., 2004). The DTC expresses at least two membrane bound DSL family ligands, LAG-2 and APX-1, which activate the GLP-1 (Notch family) receptor on nearby germ cells. Downstream, GLP-1 acts via LAG-1 to inhibit the accumulation of specific RNA-binding proteins, preventing meiotic entry (reviewed by Hansen and Schedl, 2013; Kershner et al., 2013).

Many system-level questions about the germ line remain unanswered. For example, what is the precise interplay between GLP-1 activity, cell cycle and meiotic entry? What are the properties of the germ cell cycle, and how do these alter with age and environmental conditions? Given that the two known DTC-expressed ligands are membrane bound, what determines when and where a germ cell enters meiosis? How does gonad structure affect germ cells, and how do germ cells, in turn, influence gonadogenesis? *In silico* models provide a complementary approach to laboratory experiments for investigating these questions.

Several previous models of the *C. elegans* germ line have been published. Setty et al. (2012) presented a 2D model of a lengthwise section through the adult gonad, with germ cells represented by circles restricted to an underlying lattice. The behavior of each germ cell in response to stimuli was modeled using a statechart – a visual formalism similar to a state machine or flowchart that specifies (1) the possible states of a cell, (2) the allowed transitions between states and (3) the conditions under which these occur (Harel, 1987). The Setty et al*.* model accurately reproduced mutant phenotypes and provided predictions concerning proliferative zone stability that were experimentally validated. Beyer et al. (2012) modeled a similar 2D section through the adult gonad using off-lattice cell mechanics. Off-lattice models have no underlying grid, and cells are allowed to move freely in space according to the force applied by their neighbors.

Here we present a combined ‘mechano-logical’ model of the germ line that incorporates new *in vivo* measurements and extends previous work in important ways. First, our approach combines a statechart description of germ cell behavior with 3D cell mechanics. Second, we take into account the rachis. Third, we cover both larval development and adult homeostasis in a single simulation. We found that introducing two new hypotheses into the model greatly facilitated agreement with experimental measurements. The first was gonadal ‘stretching’ during late L4. The second was mechanical feedback on adult germ cell proliferation akin to contact inhibition. We examined a scenario in which distal cells become more tightly packed, and obtained results consistent with a contact inhibition mechanism *in vivo*. Finally, we incorporated the means to perform cell tracking and clonal labeling *in silico*, to facilitate interpretation of future experimental results.

## RESULTS

### Model overview

We begin by summarizing the model (see Materials and Methods and supplementary materials and methods for further details).

**Fig. 2.**
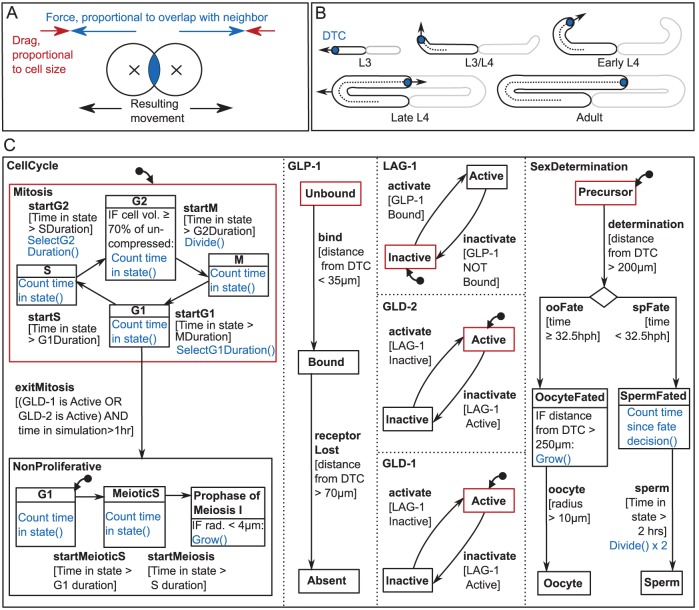
**The computational model.** (A) Cell mechanics. Overlapping cells experience a repulsion force, and the net force on a cell along with its drag coefficient determines movement at each time step. (B) Growth of the gonad boundary. The DTC (blue) migrates in a U-shape, turning at a prescribed time. The boundary at time *t* consists of all points a distance *r(t)* from the DTC path, where *r(t)* is the gonad radius. Germ cells are restricted to this domain. The model simulates a single gonad arm; the other arm is outlined in gray. (C) The statechart governing germ cell behavior. Dotted lines separate the orthogonal regions of the chart, which deal with different processes and update simultaneously. Each box represents a cell state and arrows indicate the allowed transitions between them. Alongside each transition in square brackets is the condition for it to occur. Red states are initially active in cells at the start of the simulation. *Mitosis* has no initial state, because cells begin the simulation in a randomly chosen cell cycle phase. Daughter cells inherit their parent's state upon division. Blue text indicates actions that occur on transition or while in a certain state. The biological hypotheses this statechart represents are discussed in the text.

The germ line was simulated using off-lattice mechanics, such that cells move freely under the influence of applied forces rather than being confined to boxes in an underlying grid. Individual germ cells were modeled as spheres, with a repulsion force exerted whenever two cells overlap (Fig. 2A). The approach is similar to that of Dunn et al. (2013); see also supplementary materials and methods. We used an existing modeling library, Chaste (Cancer Heart and Soft Tissue Environment) (Mirams et al., 2013), to which was added new code to model the growing gonad and to allow the use of statecharts. Code for both new features is freely available under an open source license (see Materials and Methods).

We simulated a single gonad arm, as both arms are symmetric. Germ cells were confined by a ‘boundary condition’: a surface representing the gonad membrane that cells cannot cross. The boundary is tube-shaped and was updated over time, forming along the path of the DTC. The DTC itself was explicitly modeled and migrated along a prescribed path over the course of the simulation (Fig. 2B). To ensure continued contact between proliferating germ cells and the migrating DTC (Starich et al., 2014), the DTC makes migratory progress only when germ cells abut the DTC. If DTC migration outpaced germ cell proliferation by one cell diameter (a situation that was minimized by our choice of larval proliferation rate) DTC migration paused temporarily. Our strategy of linking boundary growth to the DTC path allows changes in DTC migration to affect gonad morphology. For instance, if DTC migration is delayed during L3 owing to inadequate germ cell proliferation, the turn still occurs at the same prescribed time in the simulation, but closer to the center of the animal, consistent with experimental observations (e.g. Austin and Kimble, 1987; Killian and Hubbard, 2004).

To determine the proper gonad size and growth rate, measurements were obtained from micrographs of larvae at various stages of development. The measured animals were grown at 20°C with abundant food; these are the conditions our model aims to reflect. A target DTC migration rate was calculated for each simulation stage (Fig. S1). The same measurements also revealed that the proximal gonad lengthens significantly during L4, growth that cannot be captured by moving the DTC alone. This gonad ‘stretching’ (discussed below) was included in the model by gradually moving the boundary turn away from the center of the animal in late L4. Finally, the rachis was taken into account by forcing cells distal to and within the turn to lie just inside the boundary edge, creating a space corresponding to the rachis in the center of the arm. Gonad diameter increased appropriately over time, based on our measurements.

A statechart associated with each cell controlled fate decisions and cell cycle progression (Fig. 2C; supplementary materials and methods). Statecharts are a visual method for specifying the behavior of complex systems (Harel, 1987). Similar to a set of multilayered flowcharts, they contain states (boxes) that can be either active or inactive. Transitions (arrows) dictate how the currently active state is allowed to change. Next to each transition is a condition (in square brackets) that must be met for it to occur.

At the start of the simulation, 16 germ cells are present and their active states are those outlined in red in Fig. 2C. At each subsequent time step, the statechart is checked to see whether conditions exist allowing a transition out of any current state. If so, that transition happens immediately and the state changes. Transition conditions may include dependencies, allowing state changes in response to a cell's position in the gonad or the time post-hatching. When a cell divides, it inherits its parent's state. See Movie 1 and supplementary materials and methods for further details on model progression.

Our statechart incorporates several hypotheses about how germ cells react to their environment. First, cells born sufficiently close to the DTC are proliferative, and they and their daughter cells may not enter meiosis within 70 µm (∼13 cell diameters) of the distal tip. Beyond this point, cells irreversibly enter meiosis at the earliest opportunity, within the cell cycle constraints described below. Seventy microns is the approximate distance at which initial meiotic entry occurs in the mid-L3; therefore, this approach represents a simple spatial threshold model of DTC signaling. We further specified that commitment to meiotic entry must occur during G1; if not, germ cells undergo one further division. Recent studies, the results of which were not available during our model building, demonstrate experimental support for a very similar general mechanism (Fox and Schedl, 2015). Fox and Schedl (2015) show that cells in mitotic S or G2 complete one final round of division before entering meiosis, indicating that the meiotic entry decision must occur before pre-meiotic S phase. As G1 is very short in this system, these results further imply that the decision to enter meiosis occurs in the previous cell cycle. However, the experimental results do not preclude the possibility that the meiotic entry decision occurs during the very short G1 of the same cell cycle, as we have modeled here. Nevertheless, consistent with the results of Fox and Schedl (2015), the mechanism modeled here links the meiotic entry decision to GLP-1 activity and to the cell cycle, such that cells experiencing low GLP-1 activity divide once, then enter meiosis. The only exception would be very rare cases where a cell is in the short G1 exactly when GLP-1 activity drops, in which case the cell would enter meiosis from G1 without an intervening division.

The sperm/oocyte decision is simply triggered by distance from the DTC (200 µm). Cells arriving at this position before a threshold time become sperm-fated, whereas cells reaching it later are oocyte-fated. Finally, for reasons discussed below, mechanical feedback akin to contact inhibition was included in the cell cycle model of adult germ cells. This feedback was applied by transiently arresting progress through the cell cycle in cells compressed to <70% of their rest volume (see supplementary materials and methods). The remainder of the results section explores the impact these hypotheses had on the simulated germ line.

### Reproducing essential features of germline development

**Fig. 3.**
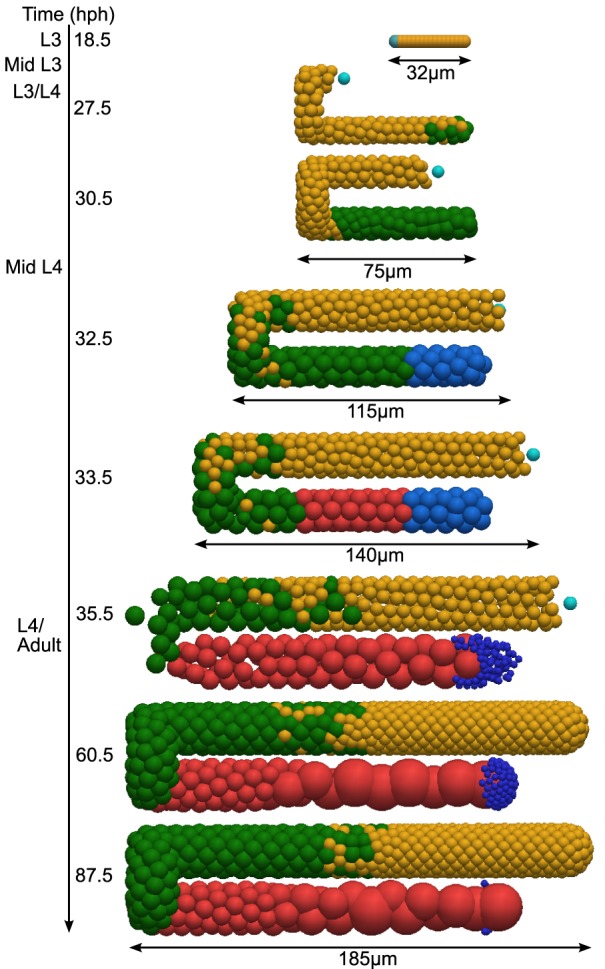
**Germline simulation snapshots, captured throughout larval development and at two adult time points.** In this simulation, germ cells in the *Mitosis* and *MeioticS* states are represented in yellow, and cells in *Prophase of Meiosis I* state are green. Total cell counts for this particular simulation are provided in Table S2. Oogenic and spermatogenic cells are represented in red and pale blue respectively, and mature sperm are dark blue. The DTC nucleus, where visible, is cyan. A proportion of oogenic cells undergo programmed cell death (not visible in these snapshots). Parameters as in Table S1, see also Movie 1.

Using the parameters set in Table S1, our simulations qualitatively resemble the wild-type germ line throughout development and into adulthood. Fig. 3 shows a series of simulation snapshots captured at key points (see also Movie 1 and Table S2). They show that the model reproduced the overall structure of the germ line: a proliferative zone was established, meiotic cells appeared at the appropriate time, sperm and oocytes were produced in roughly correct numbers, and the organization of the germ line was stably maintained for several days. Importantly, we were able to achieve these results using biologically reasonable parameters and gonad dimensions, and without biasing cell movement with respect to the DTC as in previous work (Setty et al., 2012). Notably, the direction of germ cell movement naturally reversed in the simulation after DTC migration was complete.

**Fig. 4.**
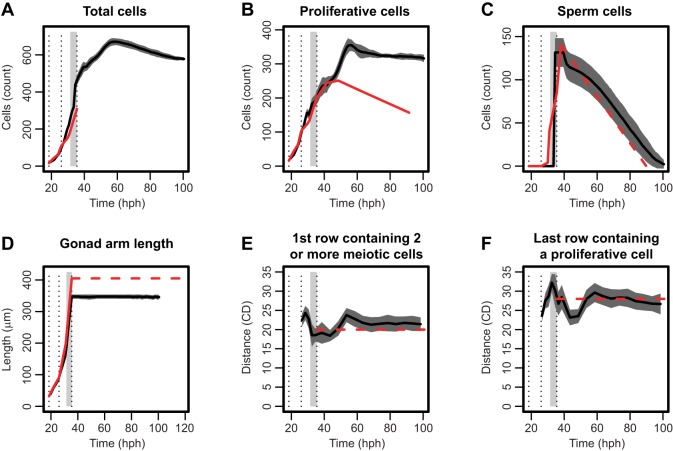
**Comparing simulated and observed values for various germline properties.** Experimental measurements are plotted alongside simulated values for several properties. In each graph, a solid black line gives the simulated result (mean of 30 runs with a region ±1 s.d. shaded), and red lines represent experimental data. Solid red lines correspond to time series and dashed lines to a single value found in the literature. Three vertical dotted lines mark the beginning of L3, L4 and adulthood. Finally, a light gray bar indicates the period where our model switches from larval to adult behavior and parameters. (A-C) Counts of total germ cells, proliferative cells and sperm, respectively, over time in hours post-hatch (hph). (D) Total length of the gonad arm in microns (µm). (E) Distance in cell diameters (CD) to the distal-most row containing two or more meiotic cells. (F) Distance in CD to the proximal-most row containing a proliferative cell. Experimental data taken from the literature (Killian and Hubbard, 2005; Hansen et al., 2004; McCarter et al., 1999) and our own measurements, including those used to generate the animation by Stupay and Hubbard on Wormatlas (http://www.wormatlas.org/hermaphrodite/germ%20line/Germframeset.html).

We fit simulation output to quantitative experimental data across a range of properties (Fig. 4). Parameters that could not be determined from the prior literature or from our own measurements are indicated by an asterisk in Table S1 and were varied during fitting within estimated feasible ranges. In general, a good agreement was achieved between simulated and expected cell counts during development (Fig. 4A-C). The counts of total cells, proliferative cells, and sperm all increased appropriately, and proliferative cell numbers were stably maintained in the adult (we did not attempt to capture the effects of ageing; Qin and Hubbard, 2015). Moreover, a steady ovulation rate was established, indicated by a falling sperm count (Fig. 4C). The length of the simulated gonad over time also matched the expected growth curve (Fig. 4D). This need not have been the case, because DTC movement can be delayed in our model if germ cells numbers are insufficient to support migration. The implication is that the rate of germ cell proliferation used here (a cycle length of 3 h in larvae and 8 h in adults) is reasonable to support normal gonadogenesis. The exact length of the germ cell cycle has been controversial in the field (Kipreos et al., 1996; Crittenden et al., 2006; Fox et al., 2011; Fox and Schedl, 2015).

There is also a reasonable fit for the length of the ‘proliferative zone’ in CD from the DTC – an estimate of the size of the stem/progenitor pool. The field defines the proliferative zone or mitotic region as the area distal to the first row containing two or more meiotic cells (Fig. 4E). We also benchmarked the proximal-most row containing a non-meiotic cell (Fig. 4F). Both properties are close on average to the expected values for the young adult. In addition, Fig. S2 compares simulated and experimental proliferative zone lengths in microns. Although the fit could perhaps be improved further with fine parameter adjustments, the current fit was considered sufficient to begin investigating overall behavior.

### Gonadal stretching during late L4 probably contributes to gonad morphogenesis

Organ growth is influenced by multiple factors and determining their relative contribution is experimentally challenging. Two independent factors contribute to gonad growth in *C. elegans*: DTC migration and the force generated by germ cell proliferation (Kimble and White, 1981; Killian and Hubbard, 2005). Previous studies have associated the force generated by germ cells with anterior-posterior extension of the gonad arms during L2 and L3.

**Fig. 5.**
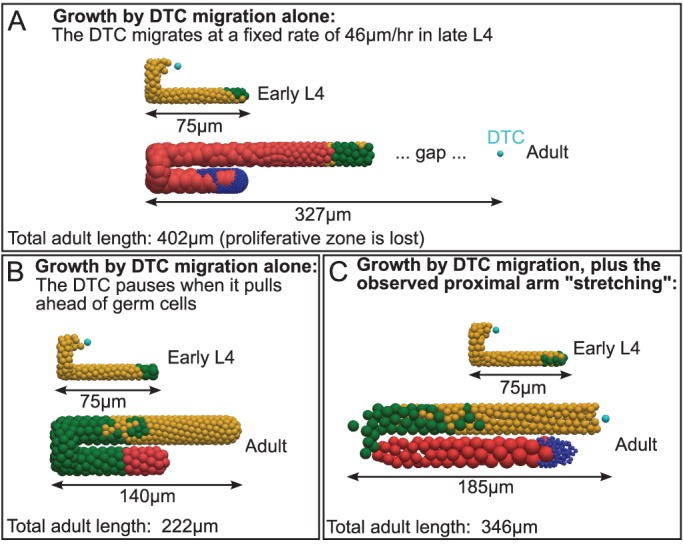
**Gonadogenesis by DTC migration alone produces unrealistic simulations.** (A) The outcome of a simulation in which gonad growth occurs by rapid DTC migration alone (no stretching). This results in the loss of the proliferative zone, as the DTC migrates faster than the germ cells and leaves a gap behind it. (B) The same simulation as shown in A with DTC pausing (see text), resulting in a shortened adult gonad, as the DTC must frequently pause until germ cells fill the available space. (C) A simulation including proximal ‘stretching’ in addition to DTC migration, resulting in growth more consistent with measurements *in vivo*.

Our experimental measurements highlighted an additional feature of larval gonadal growth. The proximal region of the gonad arm doubles in length during late L4, as the turn moves away from the center of the animal (Table 1, proximal region data). We specified this stretching in our model (Movie 1). Centrifugal growth of the proximal gonad over this period is difficult to attribute to DTC movement. To investigate whether a reasonably sized gonad could be produced by DTC migration alone, we ran a simulation with proximal stretching disabled. We calculated that without stretching, the DTC must travel at 46 µm/h during late L4 to reproduce normal growth. However, in a simulation using this migration rate, the DTC pulled ahead of distal germ cells (Fig. 5A). We therefore ran a second simulation using the same migration rate, but forcing the DTC to pause whenever it pulled ahead of distal germ cells. This alternative modeling choice maintained the proliferative zone, but produced a shortened adult gonad (Fig. 5B). By contrast, when proximal ‘stretching’ was included in the model by moving the turn, simulations produced a reasonably sized gonad, while keeping the organ filled with germ cells as *in vivo* (Fig. 5C). We conclude that elongation of the proximal region during late L4 is a non-negligible component of normal gonadogenesis. We speculate that germ cells exert pressure on the turn, forcing the gonad to ‘stretch’ during late L4.

**Table 1.**
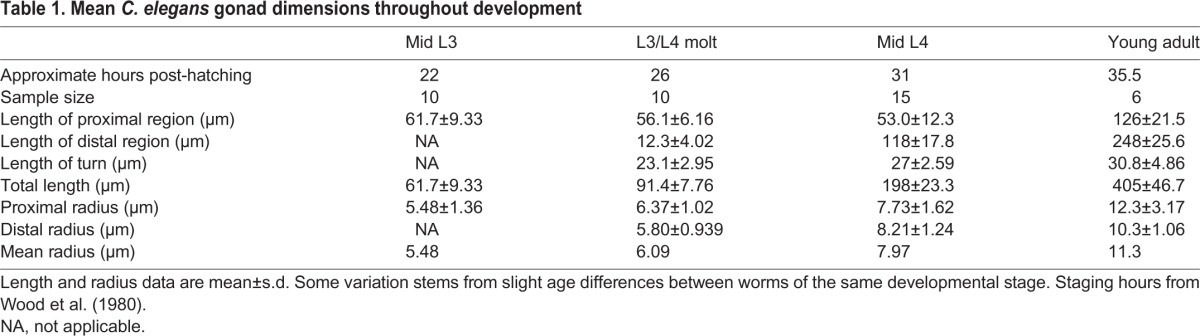
**Mean *C. elegans* gonad dimensions throughout development**

### Adult proliferative zone homeostasis might require mechanical feedback

**Fig. 6.**
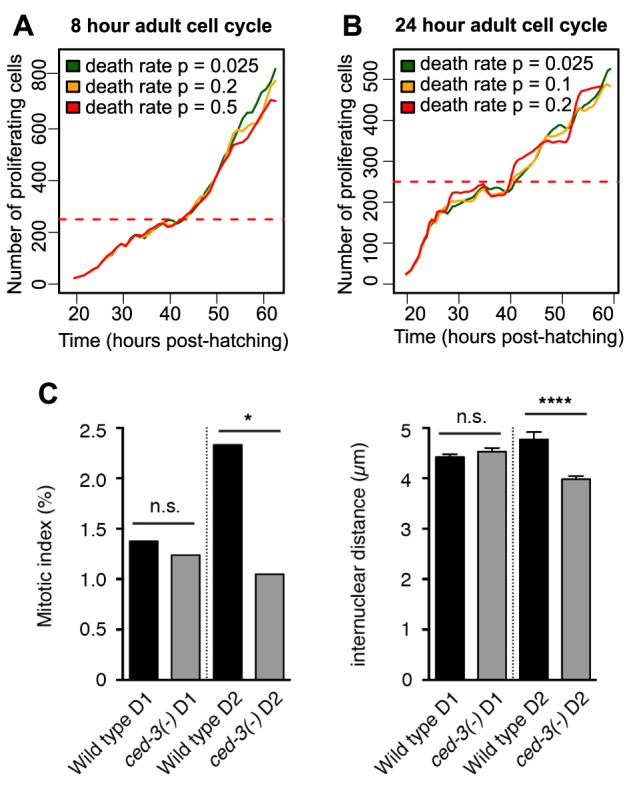
**Strategies for stabilizing proliferative cell numbers.** (A,B) Attempts to balance rates of cell proliferation and death in the absence of mechanical feedback. Both panels show the number of proliferative cells in the simulation over time for three different values of the death rate (mean of five runs). Horizontal red dashed lines indicate the expected proliferative cell count. (C) Left: a comparison of proliferative zone mitotic index in wild type and in the *ced-3(n717)* mutant at 1 and 2 days post L4 (D1 and D2, respectively). Mann–Whitney U test, * *P<*0.05. Right: internuclear distance index as calculated from the same animals. Two-tailed Student's *t*-test, ****P<0.0001. The number of gonad arms analyzed: 33 (N2 D1), 23 [*ced-3(-)* D1], 22 (N2 D2), 21 [*ced-3(-)* D2]. n.s., not significant. Error bars represent standard error of the mean, s.e.m.

In the course of model building, maintaining a stable number of proliferative cells in the young adult proved problematic. Initially, we tried to achieve cell number homeostasis by balancing a fixed adult cell division rate with a fixed death rate. Fig. 6A,B demonstrates how this approach failed. These simulations always produced an unrealistic explosion in the number of proliferative cells, even when the death rate was increased to the extent that ovulation was impaired. No germ cell death rate in this range was sufficient to balance a cell cycle length of 8 h. The same was true for a longer cell cycle length of 24 h (the longest estimate in the literature; Crittenden et al., 2006). We also tried introducing mechanical feedback on cell death by making heavily compressed germ cells more likely to undergo apoptosis. However, this caused ovulation to halt following excessive cell death. Of the mechanisms we have simulated thus far (changing rates of proliferation, of movement as influenced by cell-cell repulsion, and of cell death), negative feedback from compression on germ cell proliferation rates provided the most robust homeostasis.

If mechanical feedback on the cell cycle were to occur *in vivo*, one prediction is that tighter packing of cells in the proliferative zone should slow the cell cycle. We sought an experimental means to induce tighter packing without manipulating key signaling pathways that regulate the size, number or fate of cells in the zone, and then determined whether the mitotic index of these more densely packed cells was altered. We examined a *ced-3* mutant in which oogenic germ cells fail to undergo physiological cell death. We reasoned that in this mutant, the larval germ line should develop normally, but that the accumulation of ‘un-dead’ cells in the loop region might pose a barrier to germ cell movement and thereby cause compression of distal cells. Indeed, at day 2 we found a lower internuclear distance among cells in the proliferative zone in the *ced-3* mutant as measured along the long (distal-proximal) axis (Fig. 6C; we found a similar internuclear distance difference along the short axis of the gonad, whereas the number of cells in the proliferative zone was not significantly different). We then measured mitotic index and found that although it is indistinguishable from wild type early in adulthood (Fig. 6C, day 1), it is significantly reduced relative to wild type after one day of oogenesis (Fig. 6C, day 2), correlating with greater compression and accumulation of ‘un-dead’ cells. These results are consistent with the possibility that tightly packed cells might cycle more slowly in response to local mechanical force.

### *In silico* cell tracking and clonal analysis

Live cell tracking and live/fixed-sample cell lineage analyses are experimental gold standards in developmental and stem cell biology, but these techniques have not yet been implemented for the *C. elegans* germ line. Long-term live cell tracking is difficult owing to germline sensitivity to stress conditions, but recent advances in germline transgene expression (Zeiser et al., 2011) may facilitate lineage analysis. Nevertheless, analysis of labeling data in the *C. elegans* germ line is complicated by fast gonad growth and the relatively short time the system remains in homeostasis (Killian and Hubbard, 2005; Qin and Hubbard, 2015). *In silico* models represent a complementary tool for testing hypotheses about germ cell dynamics, providing predictions for the corresponding experiments. We therefore incorporated cell tracking and lineage recording capabilities into our model.

**Fig. 7.**
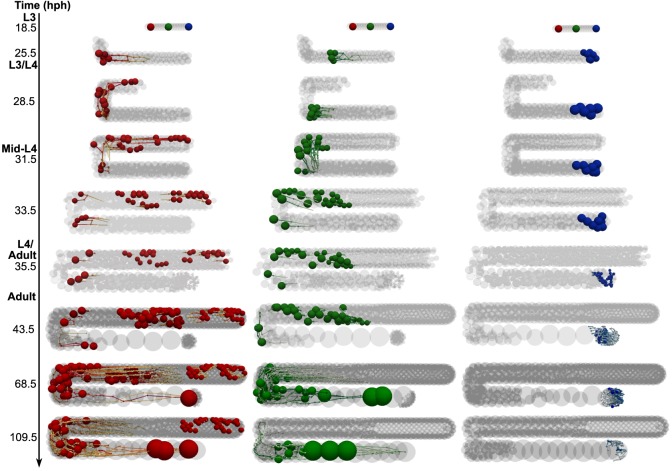
**Tracking individual germ cells.** In this simulation, three cells were labeled at the beginning of L3: one distal (red), one mid (green) and one proximal (blue). The three columns of the figure show the paths taken by each of these cells, along with their descendants. Other germ cells are indicated in light gray. Note that some tracks do not end in a cell, owing to removal from the simulation by apoptosis or ovulation; this is particularly apparent at 109.5 hph, as green oocytes are ovulated.

First, the paths taken by a small sample of germ cells and their descendants were traced (Fig. 7). Three cells were labeled in early L3, one from each of the distal, mid and proximal regions of the gonad arm. The simulation showed that descendants of a single cell remained tightly grouped initially. During L3, the dominant direction of cell movement was towards the DTC; this reversed in adulthood (Fig. 7, 35.5-43.5 h; Movie 1). Going into L4, the distal-most cells (red) traveled further and faster than proximal cells, filling the space created by DTC migration. The distal-most cells also produced more descendants, because they remained longer in the proliferative zone during gonadogenesis. Meanwhile, proximal cells (blue) remained pressed into the proximal end and eventually developed into sperm. Later in L4, all clonal groups became more dispersed, particularly in the mid-gonad. Some germ cell movement towards the turn also occurred over this period, as cells moved to fill the space created by stretching (Fig. 7, 33.5 h post-hatching).

Tracking into adulthood showed that the descendants of the proximal-most cells at L3 were eliminated from the gonad by apoptosis, or ovulation and fertilization (note the few remaining green and blue cells at the final time point). However, multiple ancestor labels remained in the simulated germ line when self-sperm were depleted. This observation is of interest with regard to the possibility of achieving monoclonality, in which all adult cells descend from a single-labeled ancestor. Monoclonality indicates a neutral drift maintenance mechanism (Snippert et al., 2010). Although not explicitly required, our model allowed neutral drift to occur. However, the lineage tracing we implemented suggests that to observe monoclonality experimentally in *C. elegans*, ovulation would need to be prolonged beyond the time of self-sperm depletion (e.g. by mating).

**Fig. 8.**
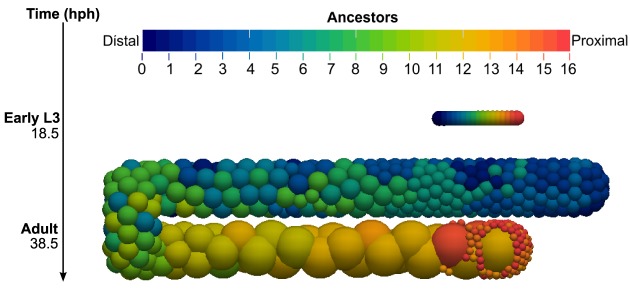
**Labeling all young adult germ cells by their L3 ancestors.** All germ cells present at the start of L3 were assigned a color that was inherited by daughter cells. The lower image shows the resulting pattern of cell ancestor labels in the adult simulated germ line.

To provide an overview of the motion and arrangement of all germ cells, we ran a simulation labeling every cell according to its L3 ancestor (Fig. 8). It confirmed that the distal-proximal position of cells is roughly maintained throughout a development simulation. Cells near the DTC at L3 contributed to the adult proliferative zone, whereas proximally positioned cells at L3 became self-sperm. Clonal groupings of cells appear at the distal end in this simulation, whereas considerable mixing occurs in the mid-gonad. It remains to be seen whether these predictions will match experimental observations, once techniques become available for *in vivo* cell tracking.

## DISCUSSION

Our current model of the *C. elegans* germ line builds on previous work and suggests new avenues of investigation. Germ cells are simulated in 3D using a combination of off-lattice cell mechanics and a logic-based response to signaling. Modeling the germ line in 3D, as well as the gonad architecture and rachis, provides a more realistic simulation. The addition of mechanics provides physical interactions among cells and naturally recapitulates germ cell movement over time, most notably a general reversal in early adulthood. The model also enforces a leader cell boundary condition, parameterized using new experimental measurements. By this approach, we obtained reasonable agreement between simulation and a range of experimental data.

The introduction of two hypothesis-generating effects not yet described in the literature greatly enhanced the fit: ‘stretching’ gonad growth during L4 and a contact inhibition-like mechanism among adult germ cells. In addition to the rapid germ cell proliferation that contributes to early anterior-posterior gonad growth (Killian and Hubbard, 2005), our model suggests that the whole organ is affected by germ cell pressure during late L4, leading to stretching and centrifugal movement of the turn. We could not produce a realistic model without incorporating this mechanism.

An exciting prediction made by the model is that adult germ cells may be subject to mechanical feedback similar to contact inhibition. Contact inhibition is a common assumption in cell-based computer models (Buske et al., 2011; Galle et al., 2005; Dunn et al., 2012), but other means of stabilizing cell numbers in a germline simulation exist. For instance, germ cells could be made highly incompressible. However, the repulsion force between cells in our simulation is already stronger than in similar cellular models (Meineke et al., 2001), and changing the strength of cell repulsion would also alter the ovulation rate, an aspect of the current model that fits the data well. Another possibility is that as yet uncharacterized mechanisms controlling the rate of entry into or progression through meiotic prophase could counter the accumulation of proliferative germ cells.

Mechanical feedback on proliferation is an attractive hypothesis. In addition to being a robust mechanism for homeostasis during the maintenance phase, it could explain why germ cells cycle faster during larval expansion and slow after gonad growth stops. Remarkably, we found that denser packing correlated with a reduced proliferation rate, as predicted. Though this correlation is consistent with a mechanical feedback mechanism, additional experimental tests of this hypothesis are needed. Contact inhibition is a major tumor suppressor mechanism (Hanahan and Weinberg, 2011), but its role as a regulator of normal tissue development and homeostasis is not well understood. If our prediction is borne out, the *C. elegans* germ line could provide a powerful *in vivo* model. Future work would also have to account for the apparent violation of this mechanism among mutants such as *glp-1(gf)* (Maciejowski et al., 2006). One attractive candidate mediator is the Hippo signaling pathway (Edgar, 2006). Components exist in the *C. elegans* genome (Yang and Hatta, 2013) but their role in germline development has not been explored.

Two other computational models of the *C. elegans* germ line appeared while this work was in revision (Hall et al., 2015; Chiang et al., 2015). Hall et al. (2015) also combines 3D, mechanically driven cell movement with a logical model of cell behavior. A major difference is that our model simulates both larval and adult stages, whereas Hall et al. simulate the adult germ line only (including genes that control oocyte maturation and cell death). Interestingly, both models found it necessary to limit mitotic growth by introducing negative feedback from pressure on germ cell proliferation. Also, neither model produced a monoclonal germ line in labeling simulations. A more detailed comparison of our work with that of Hall et al. (2015) will require access to their code and additional details regarding the qualitative network model. By contrast, Chiang et al. (2015) takes a lattice-based approach to germ cell movement, and focuses on identifying cell cycling strategies that minimize the accrual of mutations while still allowing for rapid expansion of the germ cell pool.

As our work represents a first-generation 3D mechano-logical model of the germ line, there remain ways in which our simulations fall short of the real system. For instance, the turn in our simulations is somewhat crowded, containing a cluster of germ cells rather than a queue of cells of increasing size. This might be remedied in future by incorporating local narrowing of the gonad turn boundary, or prescribing flow from the rachis or other signals to regulate oogenic cell growth (Wolke et al., 2007; Nadarajan et al., 2009; Govindan et al., 2009).

In general, long simulation run time has hindered our ability to explore the full parameter space of the model systematically. Thus, another choice of parameters may yet exist producing a better fit to data. We have, however, varied the unconstrained parameters about their current values (Figs S4-S9) and varied two key parameters simultaneously (adult cell cycle length and cell-cell repulsion; Fig. S10). This analysis suggests that larval cell cycle time bears on multiple aspects of germline development, consistent with previous findings (Killian and Hubbard, 2004; Michaelson et al., 2010; Korta et al., 2012, Setty et al., 2012). Variations in this parameter impacted gonad growth, sperm count, proliferative zone accumulation and meiotic entry. These effects contrast with variation in other experimentally undetermined parameters that do not alter germline growth dynamics. Although variation in the position at which sex determination occurs affects sperm count (and, modestly, the proliferative zone owing to compression changes), we note that the distance-based implementation of this feature is somewhat artificial. Therefore, these impacts should be revisited when a more biologically based mechanism for sex determination is implemented, such as coupling to meiotic entry (Barton and Kimble, 1990).

Finally, we have simplified the system in certain respects to make it more tractable. For example, although we took into account radial gonad growth, local alterations in radius at the distal tip and turn are neglected. This might have a bearing on the fit between cell row counts and micron measurements of the proliferative zone. Despite these areas for improvement, the model provides a strong starting point on which to build in future work.

Future investigations using this model could also further develop the cell statechart. For example, it will be of interest to challenge the model under conditions that interfere with *glp-1* activity and/or cell cycle progression. In addition, the impact on gonadogenesis and cell dynamics of mutations affecting cell size could be studied *in silico* (Arur et al., 2009; Korta et al., 2012). Finally, the modeling approach described here could be applied to other developmental systems, such as intestinal organoids (Sato et al., 2009).

To conclude, we have developed and tested a detailed *in silico* model of the *C. elegans* germ line, that combines, for the first time, a 3D mechanics simulation with a statechart model of cell behavior to generate new predictions. These include a role for germ cell pressure in multiple aspects of gonadogenesis and possible mechanical feedback on the adult germ cell cycle.

## MATERIALS AND METHODS

### Cell mechanics and boundary conditions

The mechanics simulation uses Chaste, an open source biological modeling library (Mirams et al., 2013). Germ cells were represented as deformable spheres, and a gonad boundary was imposed that follows DTC migration. Please see supplementary materials and methods for details on both of these features.

### Intracellular model and progression during a simulation

Whereas the mechanics simulation governs cell movement and death, in tracellular aspects of the model are governed by a statechart (Fig. 2C; supplementary materials and methods). The statechart contains orthogonal regions controlling the cell cycle, response to DTC signaling, and sex determination. We describe below how each region acts during a simulation.

Germ cells present at the start of the simulation are in the initial states colored red in Fig. 2C. Thereafter, daughter cells inherit their parent's state.

In the *CellCycle* region, cells are initially in *Mitosis* and cycle through the phases G1, S, G2 and M, dividing on entry into M phase. The transition out of a phase occurs after a certain time delay. To avoid synchronized cell cycles, the durations of G1 and G2 are sampled from a normal distribution, with mean *µ* and standard deviation *sµ* (*s*=0.1). *s* is a stochasticity parameter, and *µ* is the expected phase length. Expected phase lengths are given in Table S1 and increase between mid-L4 and adulthood. A mechanical feedback mechanism is applied to adult germ cells only, which transiently arrests progress through the cell cycle whenever a cell's volume falls below 70% of its rest value. The rest volume of a cell is simply the volume of a sphere of the same radius. The compressed volume is calculated by taking into account deformation due to neighboring cells (see supplementary materials and methods). For this model, we chose to delay the cell cycle in G2, as this portion of the cell cycle is sensitive to other cues such as nutrition (Fukuyama et al., 2006; Michaelson et al., 2010). Germ cells in the model exit mitosis only when they enter G1 within a region where GLD-1 or GLD-2 is active. Therefore, cells that complete G1 (that is, are in S or G2) as they enter an active GLD region will divide once (and only once) prior to becoming *NonProliferative*. Upon exiting mitosis, a fixed length G1 and meiotic S occur before the cell enters meiotic prophase. We note that these cell cycle choices – G2 for sensitivity to compression and G1 for commitment to meiosis – can be altered in future versions of the model in response to new experimental results. Meiotic cells grow steadily, up to a maximum radius of 4 µm.

Several statechart regions deal with the response to LAG-2/APX-1. The GLP-1 receptor is initially *Unbound*. At <35 µm from the DTC, the GLP-1 receptor becomes *Bound*. LAG-1 then activates, GLD-1 and GLD-2 inactivate, and the cell remains in mitosis. At ≥70 µm from the DTC, the GLP-1 signal becomes *Absent*. LAG-1 switches off, and the GLD pathways promote meiotic entry.

In the *SexDetermination* region of the statechart, all cells begin as *Precursors*, and make a sperm/oocyte fate decision on reaching 200 µm from the DTC. If this decision occurs before 32.5 hours post-hatch (hph) the cell will be sperm-fated, otherwise it is oocyte-fated. After a short delay, sperm-fated cells divide twice to produce four sperm. Meanwhile, oocyte-fated cells grow steadily and are considered mature on reaching 10 µm in radius. For growth rates, see Table S1. Oocyte-fated cells only may be removed by apoptosis (Jaramillo-Lambert et al., 2007; Gumienny et al., 1999), which occurs with probability *P* for each hour spent outside the adult proximal gonad (<250 µm from the DTC). *P* is given in Table S1. It is assumed that oocyte-fated cells that reach the proximal gonad are committed to becoming mature gametes. At the proximal end of the oviduct, oocytes are removed from the simulation along with one sperm.

### Parameter set and software

Table S1 lists parameter values and their sources, and indicates which parameters were varied during fitting. A 100-h simulation takes 10-12 h on one core of an Intel Core i5 machine.

To reproduce our results, Chaste and its dependencies must be installed (https://chaste.cs.ox.ac.uk/trac/wiki/InstallGuides/InstallGuide). Additional files specific to this project can then be downloaded (https://github.com/Katwell/ElegansGermline). All code is covered by a three-clause BSD license; see Chaste website. The model outputs vtu files for visualization in Paraview (Utkarsh, 2015), and text files from which graphs were generated in R. Further instructions and scripts are included in the Github download.

### Experimental methods

#### Determining larval gonad dimensions

Worms were grown at 20°C with abundant food. Developmental stage was determined by vulval morphology. Differential interference contrast images of live worms were captured, and measured using ImageJ (Schneider et al., 2012). Fig. S3 shows each measurement, and Table 1 summarizes the results. In addition, 1 µm *z*-stacks were obtained from live worms containing a germ cell membrane GFP marker (*xnSi1*; Chihara and Nance, 2012) to estimate the radius of germ cells and of the rachis.

#### Mitotic index and internuclear distance

In parallel, wild-type and *ced-3(n717)* mutant worms were synchronized, ethanol fixed and DAPI stained as described by Pepper et al. (2003). Microscopy and determination of mitotic index were as described by Michaelson et al. (2010). Internuclear distance index is the distance from the distal tip to meiotic entry in microns over the distance in CD.
